# Carrier systems of radiopharmaceuticals and the application in cancer therapy

**DOI:** 10.1038/s41420-023-01778-3

**Published:** 2024-01-09

**Authors:** Taotao Zhang, Huiwen Lei, Xiaohua Chen, Zhihui Dou, Boyi Yu, Wei Su, Wei Wang, Xiaodong Jin, Takanori Katsube, Bing Wang, Hong Zhang, Qiang Li, Cuixia Di

**Affiliations:** 1 Bio-Medical Research Center, Institute of Modern Physics, Chinese Academy of Sciences, Lanzhou, 730000 China; 2 Key Laboratory of Heavy Ion Radiation Biology and Medicine of Chinese Academy of Sciences, Lanzhou, 730000 China; 3 College of Life Sciences, University of Chinese Academy of Sciences, 101408 Beijing, China; 4 School of Nuclear Science and Technology, University of Chinese Academy of Sciences, 101408 Beijing, China; 5 Advanced Energy Science and Technology Guangdong Laboratory, Huizhou, 516029 China; 6 College of Life Science, Northwest Normal University, Lanzhou, 730000 China; 7 National Institute of Radiological Sciences, National Institutes for Quantum Science and Technology, Chiba, 263-8555 Japan

**Keywords:** Targeted therapies, Radiotherapy

## Abstract

Radiopharmaceuticals play a vital role in cancer therapy. The carrier of radiopharmaceuticals can precisely locate and guide radionuclides to the target, where radionuclides kill surrounding tumor cells. Effective application of radiopharmaceuticals depends on the selection of an appropriate carrier. Herein, different types of carriers of radiopharmaceuticals and the characteristics are briefly described. Subsequently, we review radiolabeled monoclonal antibodies (mAbs) and their derivatives, and novel strategies of radiolabeled mAbs and their derivatives in the treatment of lymphoma and colorectal cancer. Furthermore, this review outlines radiolabeled peptides, and novel strategies of radiolabeled peptides in the treatment of neuroendocrine neoplasms, prostate cancer, and gliomas. The emphasis is given to heterodimers, bicyclic peptides, and peptide-modified nanoparticles. Last, the latest developments and applications of radiolabeled nucleic acids and small molecules in cancer therapy are discussed. Thus, this review will contribute to a better understanding of the carrier of radiopharmaceuticals and the application in cancer therapy.

## Facts

- Radiopharmaceuticals hold great promise for the future of cancer therapy. Effective application of radiopharmaceuticals depends on the selection of an appropriate carrier.
- The most employed carriers of radiopharmaceuticals include antibodies, peptides, nucleic acids, small molecules, and nanoparticles, each of which has advantages and disadvantages.
- The high specificity and affinity of antibodies for target antigens make them an excellent carrier for radionuclide delivery. Antibody derivatives and the use of pretargeting strategies have further improved the therapeutic efficiency of radiolabeled antibodies.
- Radiolabeled peptides are a very specific radiopharmaceutical group. There is a growing interest in the development of novel peptide carrier systems, such as heterodimers, bicyclic peptides, and peptide-modified nanoparticles.

## Open questions

- High specificity and affinity of the carrier for the target are crucial, and how can they achieve optimal?
- Despite the increasing number of radiolabeled antibodies developed in preclinical studies, translation of the most prospective agents has remained challenging.
- Radiolabeled peptides have undergone extensive preclinical studies and novel peptide carrier systems continue to be developed, but there are still challenges in their translation to clinical trials.

## Introduction

Cancer is one of the leading causes of human death worldwide [1]. Despite increasing treatment options for cancer patients, such as radiotherapy, targeted therapy, and immunotherapy, etc., there is still need for treatment optimization [2]. Radiopharmaceutical therapy (RPT) as a novel therapeutic modality for cancer, attracted a high degree of recognition and commercial interest [3, 4]. RPT delivers radionuclides to tumor-related targets, where they can inhibit or destroy tumor tissues to achieve therapeutic purposes [5]. However, few radionuclides themselves can selectively target tumor sites, such as radioiodine targeted therapy for thyroid diseases [6], most radionuclides need to be guided by carriers to reach the target tissue [4, 7]. Therefore, the study of carrier systems related to radionuclide delivery has become an important area of a significant interest and growth. To date, a large number of studies have been carried out on the carrier of radiopharmaceuticals, and new carriers are constantly being explored and discovered [3, 8, 9]. Hence, in this review, we briefly describe the carrier of radiopharmaceuticals. Subsequently, radiolabeled antibodies are outlined, as well as novel approaches for the treatment of lymphoma and colorectal cancer (CRC) using radiolabeled antibodies. Furthermore, we review radiolabeled peptides, and novel strategies of radiolabeled peptides in the treatment of neuroendocrine neoplasms (NENs), prostate cancer, and gliomas, notably heterodimers, bicyclic peptides, and peptide-modified nanoparticles (NPs). Last, the latest developments and applications of radiolabeled nucleic acids and small molecules in cancer therapy are discussed. Thus, our elucidation will contribute to a better understanding of radiopharmaceutical carrier systems and the application in cancer therapy.

## Carrier systems of radiopharmaceuticals

**Fig. 1:**
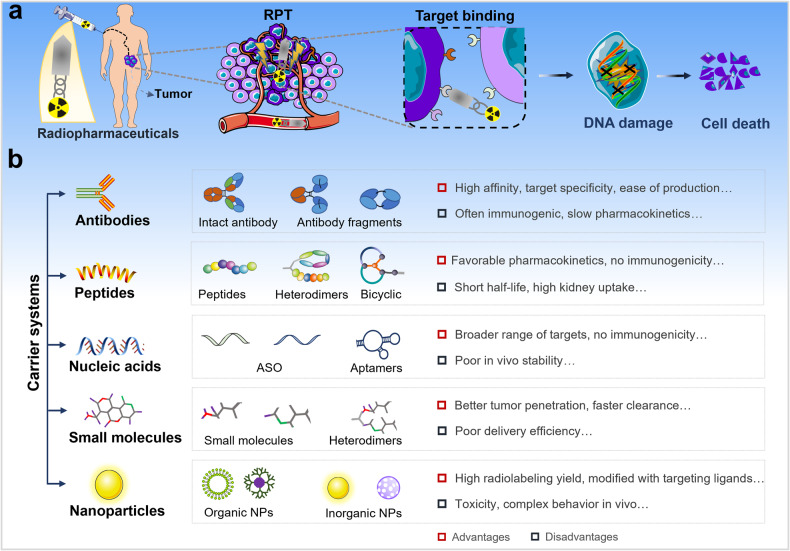
The carriers of radiopharmaceuticals and the application in cancer therapy. **a** Carriers of radiopharmaceuticals with high affinity and specificity for the tumor target can selectively guide therapeutic radionuclide payloads to tumor cells for targeted therapy. **b** The main categories of common carrier systems used for radiopharmaceutical development and advantages and disadvantages of various carriers.

Radiopharmaceuticals generally consisting of radionuclides coupled to targeting carriers [10]. The carrier is responsible for selectively interacting with the target site, resulting in a higher concentration of radionuclides at the target site [11]. The specificity and selectivity required for pinpointing cancer cells for cell killing has been made possible by targeted carriers [4], as shown in Fig. 1a. Tumor cells express one or more proteins that are not expressed or are rarely expressed in normal tissues or other diseases, which can be precisely recognized and bound by carriers to deliver radionuclides to the tumor site. The ionizing radiation emitted by therapeutic radionuclides can cause damage to cellular DNA and other functional macromolecules, inhibiting or killing cancer cells while minimizing damage to healthy tissue [12, 13]. The selection of an ideal carrier to accurately deliver the radionuclide to specific tumor cells is the basis for achieving better therapeutic effect of RPT. The requirements of targeted carriers include high affinity and specificity for the target, non-toxic or non-immunogenic, and easy to produce and modify, etc. [11, 13, 14].

Currently, the most employed carriers are antibodies, peptides, nucleic acids, small molecules, and NPs, etc., as shown in Fig. 1b. Antibodies were the first biological carriers employed for radiopharmaceuticals. The high affinity and specificity of antibodies for target antigens overexpressed on tumors make them excellent carriers for radionuclide delivery, especially monoclonal antibodies (mAbs) and their derivatives [15, 16]. However, intact mAbs have inherent main drawbacks due to their relatively high molecular weight, including slow pharmacokinetics and low diffusivity within solid tumors [17]. Although antibody fragments can improve the pharmacokinetics of solid tumor therapies, they are reduced in stability, show a significant degree of nonspecific accumulation in healthy tissues [17–20]. Peptides have many advantages that make them attractive radiopharmaceutical carriers, including ease of synthesis and radiolabeling, favorable pharmacokinetics, low toxicity and immunogenicity, etc. [21]. In particular, the development of heterodimeric peptides and bicyclic peptides provides powerful carrier systems [22, 23]. In recent years, nucleic acids are also being actively explored as carriers of radiopharmaceuticals, which have some significant advantages in terms of production, modification, possible targets, and immunogenicity, etc. [24]. Moreover, small molecule radiopharmaceuticals can rapidly penetrate tumors and be rapidly cleared from non-target tissues, etc., thus reducing toxicity, compared to large molecules [25]. This therapeutic approach is receiving increasing attention. Furthermore, the advanced hybrid radionuclide carriers are being developed, such as antibody- and peptide-modified NPs have been studied in radionuclide delivery and hold considerable promise in cancer therapy [26, 27]. This review focuses on radiolabeled antibodies, peptides, nucleic acids, small molecules and their novel strategies in cancer therapy.

## Radiolabeled antibodies in cancer therapy

### Approved radiolabeled mAbs and their derivatives

Currently, two radiolabeled anti-CD20 murine mAbs, ^90^Y-ibritumomab tiuxetan (Zevalin) and ^131^I-tositumomab (Bexxar) have been approved by the United States Food and Drug Administration (FDA) for the treatment of relapsed or refractory, low-grade or follicular B cell Non-Hodgkin lymphoma (NHL), including rituximab refractory follicular NHL in 2002 and 2003, respectively [28]. ^131^I-Metuximab for the treatment of hepatocellular carcinoma (HCC) and ^131^I-chTNT for the treatment of lung cancer have been approved by the China National Medical Products Administration [29, 30]. Clinical translation of radiolabeled antibodies therapy began with the approval of Zevalin and Bexxar. In a phase III study [31], the Zevalin RPT was compared with rituximab immunotherapy in 143 patients with relapsed or refractory low-grade, follicular, or transformed B‑NHL. Patients received a single intravenous dose of 0.4 mCI/kg of Zevalin (*n* = 73) or four weekly doses of 375 mg/m^2^ of rituximab (*n* = 70). The overall response rate for the Zevalin group and rituximab group were 80% and 56%, respectively, and complete response were 30% and 16%, respectively. Later, in a randomized phase III first-line indolent trial [32], Zevalin was shown to be highly valuable as first-line consolidation therapy in patients with advanced-stage follicular lymphoma. Among 409 patients available for analysis, estimated 8-year overall progression-free survival was 41% with Zevalin group versus 22% for control, and the median time to the next treatment was 8.1 years for Zevalin group versus 3.0 years in the control group. Additionally, in clinical trials of Bexxar, Kaminski et al. reported very encouraging results in patients with follicular lymphoma [33]. In this study, 76 patients with stage III or IV follicular lymphoma receiving Bexxar as initial treatment. Results showed that overall response rate was as high as 95%, and 75% of patients having a complete response. After a median follow-up of 5.1 years, 5-year progression-free survival was 59% and median progression-free survival was 6.1 years. These advances then opened the door widely to cancer therapy.

### Novel strategies of radiolabeled mAbs and their derivatives in cancer therapy

#### Non-Hodgkin lymphoma

**Fig. 2:**
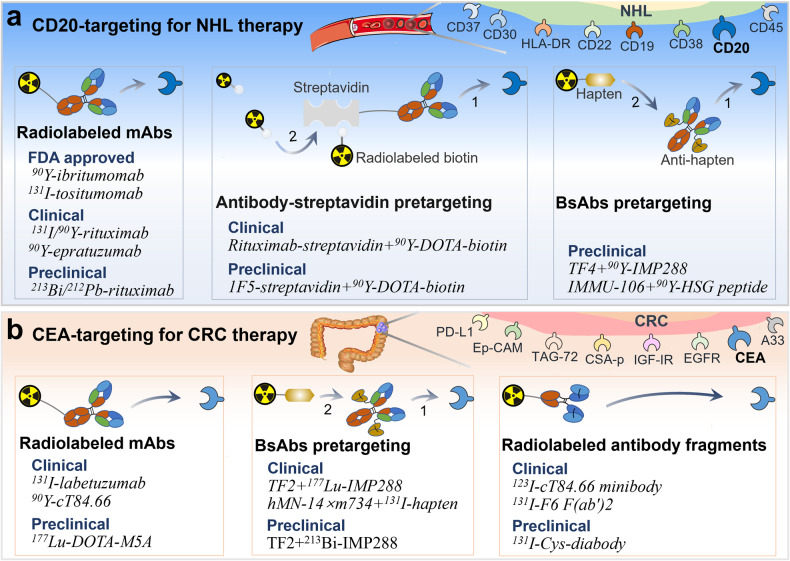
Schematic representation of radiolabeled antibodies in cancer therapy. **a** The strategy of targeting CD20 for the treatment of NHL and some corresponding radiopharmaceuticals. Major therapeutic strategies include direct radiolabeled antibodies, streptavidin-biotin pretargeting, and bsAb pretargeting approaches. **b** The strategy of targeting CEA for the treatment of colorectal cancer and some corresponding radiopharmaceuticals. Major therapeutic strategies include direct radiolabeled antibodies and antibody fragments, and bsAb pretargeting approaches.

After the first approval, a significant number of preclinical and clinical studies evaluated the efficacy of radiolabeled antibodies in lymphomas and therapeutic strategies, particularly anti-CD20 RPT (Table 1 and Fig. 2a) [34, 35]. Radiolabeled anti-CD20 chimeric mAb rituximab has large potential to be employed for NHL. Rituximab has the ability to induce antibody-dependent cellular cytotoxicity and apoptosis via intrinsic and extrinsic apoptotic pathways [36, 37]. A phase II study demonstrated that 99% of NHL patients showed a complete or partial response to ^131^I-labeled rituximab, and the treatment had low toxicity and was less expensive than chemotherapy [38]. Additionally, other novel strategies to improve the efficiency of RPT are also being developed. Pretargeted RPT is a novel promising approach aiming to improve clearance and reduce off-target toxicity, therefore minimizing toxicity and maximizing therapeutic response [39], including the streptavidin-biotin and bispecific antibodies (bsAbs) pretargeting approaches. In streptavidin-biotin method, mAbs and radioactive agents are used separately (Fig. 2a). Biotin has a higher affinity to streptavidin than the average affinity of antigen-antibody, and a streptavidin molecule can bind to multiple radioactive biotins. Based on this, higher doses of radiation can be delivered to the targeted tissue [39]. Preclinical studies have shown that streptavidin-biotin RPT has better efficacy and lower toxicity than directly radiolabeled mAbs in lymphoma xenografts [40–42]. However, streptavidin-biotin RPT is limited by its immunogenicity and interference with endogenous biotin, which may complicate the clinical translation of this approach [43]. BsAbs pretargeting approach involves the use of unlabeled bsAbs with affinity to both radiolabeled hapten and tumor-associated antigen (Fig. 2a). According to the bsAbs pretargeting approach, one arm of bsAb targets the tumor antigen and the other arm recognizes the radiolabeled hapten used for RPT [44]. This pretargeting approach improves the therapeutic response of targeted radionuclides in NHL compared to directly radiolabeled mAbs [45]. For example, Sharkey et al. reported that pretargeting method using anti-CD20 bsAb TF4 followed by ^90^Y-DOTA-peptide significantly improved survival, and cured 33% to 90% of lymphoma nude mice, even at relatively low dose [46]. Based on these studies, it can be concluded that pretargeting is a promising cancer therapy approach that can improve the efficiency of RPT. Other effective strategies are also being explored.

**Table 1:** Examples of antibody-based radiopharmaceuticals for cancer therapy.

Antigen	Cancer	Antibody	Radiopharmaceuticals	Stage	Ref.
CD20	NHL	mAb	^90^Y-Ibritumomab tiuxetan	Approved	[28]
CD20	NHL	mAb	^131^I-Tositumomab	Approved	[28]
CD147	HCC	F(ab’)_2_	^131^I-Metuximab	Approved	[29]
DNA	Lung cancer	mAb	^131^I-chTNT	Approved	[15]
CD20	NHL	mAb	^131^I/^177^Lu-DOTA/^90^Y-Rituximab	II/I/II	[38, 115, 116]
CD20	NHL	mAb	^213^Bi/^212^Pb-Rituximab	Preclinical	[117, 118]
CD20	NHL	mAb	Rituximab-Streptavidin + ^90^Y-DOTA-Biotin	I/II	[43]
CD20	Lymphoma	mAb	1F5-Streptavidin + ^90^Y-DOTA-Biotin	Preclinical	[40]
CD22	Lymphoma	mAb	^90^Y-DOTA-Epratuzumab	II	NCT00906841
CD37	NHL	mAb	^177^Lu-Lilotomab-Satetraxetan	I/II	NCT01796171
HLA-DR	Lymphoma	mAb	^131^I-Lym-1	I	NCT00028613
CD38	Lymphoma	mAb	^177^Lu-Daratumumab	Preclinical	[119]
HK2	Prostate cancer	mAb	^177^Lu-m11B6	Preclinical	[120]
PSMA	Prostate cancer	mAb	^177^Lu-J591	II	NCT00195039
CD46	Prostate cancer	mAb	^212^Pb-TCMC-YS5	Preclinical	[121]
CD33	AML	mAb	^225^Ac-Lintuzumab	I/II	NCT02575963
CD38	Myeloma	mAb	^211^At-OKT10-B10	I	NCT04466475
CD45	AML	mAb	^131^I-BC8	II	[122]
CEA	Colorectal cancer	mAb	^131^I-Labetuzumab	II	[49]
CEA	Colorectal cancer	mAb	^90^Y-cT84.66	I/II	NCT00645710
CEA	Colorectal cancer	mAb	^177^Lu-DOTA-M5A	Preclinical	[123]
PD-L1	Colorectal cancer	mAb	^131^I-Atezolizumab	Preclinical	[124]
A33	Colorectal cancer	mAb	^131^I-huA33	I	NCT00291486
HER2	Ovarian cancer	mAb	^211^At-Trastuzumab	Preclinical	[125]
HER2	Breast cancer	mAb	^211^At-AuNP-Trastuzumab	Preclinical	[26]
Ep-CAM	Colorectal cancer	mAb	^125^I-17-lA	Clinical	[126]
TAG-72	Colorectal cancer	mAb	^131^I-CC49	I	[127]
CSA-p	Colorectal cancer	mAb	^131^I-Mu-9	I	[128]
HK2	Prostate cancer	mAb	^177^Lu-hu11B6	Preclinical	[129]
CEA	Colorectal cancer	Fab’/F(ab’)_2_	^131^I-Hdfm/F6 F(ab’)_2_	I/II-II	[53, 130]
EGFR	Colorectal cancer	F(ab’)_2_	^177^Lu-DOTAGA-F(ab’)_2_-Cetuximab	Preclinical	[131]
CD22	B-NHL	Diabodies	^177^Lu-huRFB4 Db	Preclinical	[132]
CEA	Colorectal cancer	Diabodies	^131^I-Cys-diabody	Preclinical	[54]
PSCA	Prostate cancer	Minibodies	^177^Lu-DTPA/^131^I-A11Mb	Preclinical	[133]
CEA	Colorectal cancer	Minibodies	^123^I-cT84.66 minibody	Clinical	[52]
HER2	Breast cancer	Nanobodies	^99m^Tc/^188^Re-NM-02	I	NCT04674722
HER2	Breast cancer	Nanobodies	^131^I-SGMIB-2Rs15d	I	NCT02683083
HER2	Solid tumor	Nanobodies	^177^Lu-DTPA/^225^Ac-2Rs15d	Preclinical	[134, 135]
CEA/HSG	Colorectal cancer	Bispecific	TF2 + ^177^Lu-IMP-288	I	NCT00860860
CEA/HSG	Colorectal cancer	Bispecific	TF2 + ^213^Bi-IMP-288	Preclinical	[51]
CEA/hapten	CEA(+)cancer	Bispecific	hMN-14×m734 + ^131^I-hapten	I	[136]
CD20/HSG	NHL	Bispecific	TF4 + ^90^Y-IMP288	Preclinical	[46]
CD20/HSG	NHL	Bispecific	IMMU-106 + ^90^Y-HSG peptide	Preclinical	[45]
CEA	Colorectal cancer	ScFv	^131^I-CIGB-M3	I	[137]
HER2	HER2(+)cancer	Affibody	^188^Re-ZHER2:41071	Preclinical	[138]
HER2	HER2(+)cancer	Affibody	^177^Lu-ABY-027	Preclinical	[91]
HER2	HER2(+)cancer	Affibody	Z_HER2:342_-SR-HP1/^177^Lu-HP2	Preclinical	[93]

#### Colorectal cancer

Radiolabeled antibodies for the treatment of CRC have been investigated in various preclinical and clinical trials (Table 1). The most commonly targeted antigens include carcinoembryonic antigen (CEA), epithelial cell-adhesion molecule, and the colon specific antigen p, etc., as shown in Fig. 2b [47]. CEA is expressed in ~95% of CRC and is the most commonly used targeted antigen [48]. To date, a number of clinical trials have evaluated the efficacy of anti-CEA RPT (Table 1) (Fig. 2b). In 2017, a phase II study of ^131^I-labeled anti-CEA mAb labetuzumab in CRC showed that the median time to progression and overall survival for all 63 patients was 16 months and 55 months, respectively [49]. In addition, bsAb pretargeting approach has been developed for CEA targeting in CRC (Fig. 2b). For example, preclinical and clinical studies have demonstrated that pretargeting with bsAb (TF2) and radiolabeled hapten peptide (IMP288) specifically and rapidly targeted tumors, and inhibited tumor growth [50, 51]. Furthermore, radiolabeled anti-CEA antibody fragments, such as minibody [52], F(ab’)_2_ [53], and diabody [54] have also been used in therapeutic studies of CRC (Fig. 2b). Wong et al. reported that ^123^I-labeled anti-CEA cT84.66 minibody showed tumor targeting to CRC and had a faster clearance compared to intact antibodies [52]. Thus, radiolabeled antibodies have shown promising potential in CRC.

## Radiolabeled peptides in cancer therapy

### Approved radiolabeled peptides

**Fig. 3:**
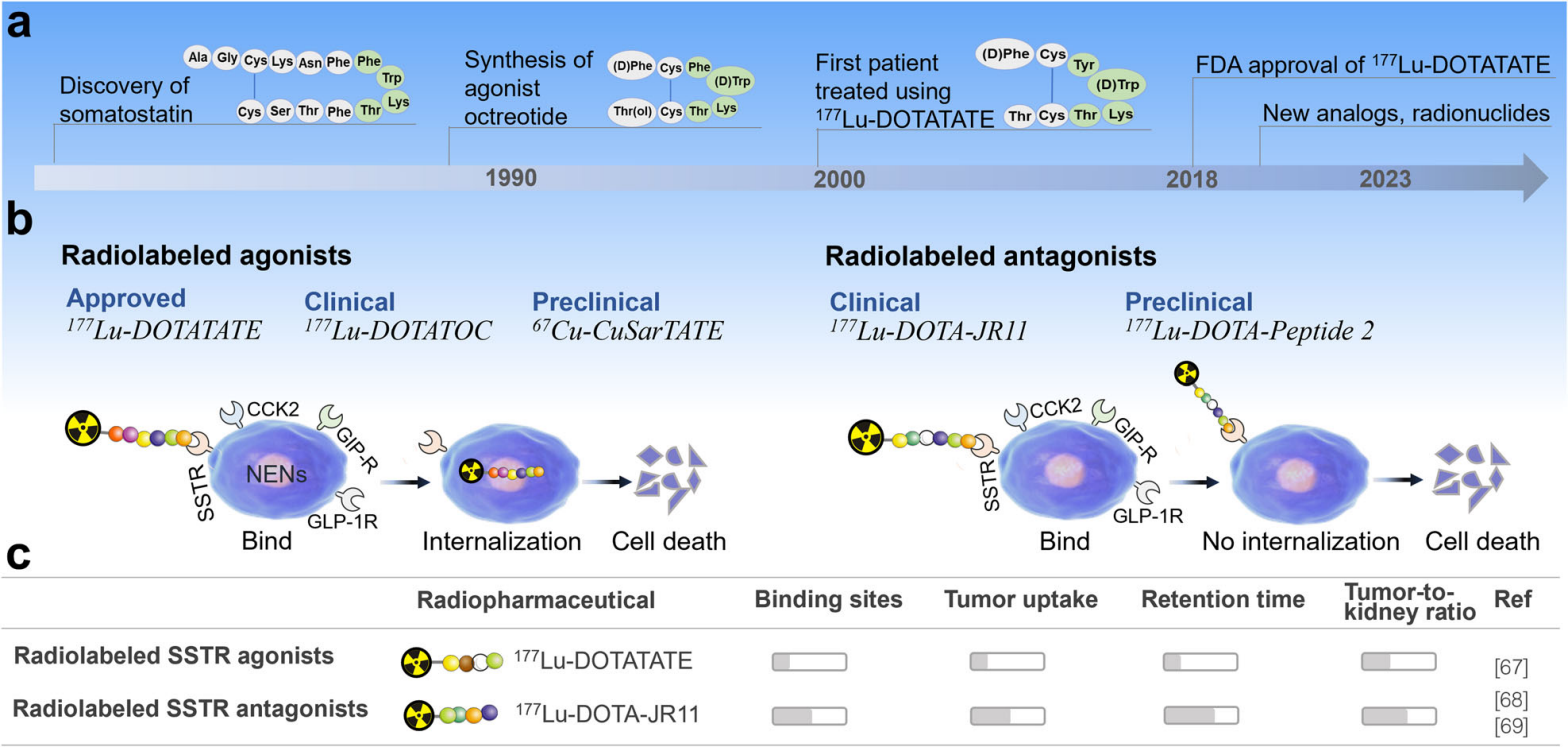
Radiolabeled peptides in neuroendocrine neoplasm therapy. **a** Development of radiolabeled somatostatin analogs for the treatment of neuroendocrine neoplasms. **b** Radiolabeled somatostatin receptor agonists and antagonists for neuroendocrine neoplasm therapy. **c** Comparison of radiolabeled somatostatin receptor agonists and antagonists in neuroendocrine neoplasm therapy.

Radiolabeled somatostatin (SST) analogs, ^177^Lu-DOTA-TATE was approved by the FDA for the treatment of adult patients with SSTR-positive gastroenteropancreatic neuroendocrine tumors (GEP-NETs) in 2018 [55]. SST is a cyclic disulfide-bond-containing peptide hormones that can bind to five somatostatin receptor subtypes (SSTR1-5) widely expressed in the whole body [56, 57]. Indeed, structure-function studies have shown that the small tetrapeptide Phe-Trp-Lys-Thr is necessary for biological activity (Fig. 3a) [58]. Recently, in the final analysis of overall survival and long-term safety of the phase III midgut NETs trial (NCT01578239) [59], the median overall survival of the ^177^Lu-DOTA-TATE group was 48.0 months and the control group was 36.3 months, which did not reach statistical significance. However, the 11.7-month difference in median overall survival with ^177^Lu-DOTA-TATE might be considered clinically relevant, and no new safety signals were reported at the time of the final analysis. Interestingly, Wu et al. showed that in a human xenograft tumor model of NET, ^177^Lu-DOTATATE RPT led to increased infiltration of CD86+ antigen presenting cells and CD49b+/FasL+ NK cells in tumor tissues [60]. Therefore, further investigation of the immunomodulatory role of RPT will be essential to improve the efficacy of cancer therapy. Undoubtedly, the clinical success of radiolabeled SST analogs has paved the way for novel peptide derivatives.

### Novel strategies of radiolabeled peptides in cancer therapy

#### Neuroendocrine neoplasms

NENs overexpress specific peptide receptors, particularly somatostatin receptors (SSTRs) (Fig. 3b) [61]. Radiolabeled somatostatin (SST) analogs targeting SSTRs are the most advanced radiopharmaceuticals for NENs (Table 2 and Fig. 3) [62, 63]. There are two major categories of SST analogs: agonists and antagonists (Fig. 3b) [63]. The agonist octreotide was the first synthesized SST analog, and was the starting point for the development of radiolabeled SST analogs (Fig. 3a) [64]. The two prevalent radiolabeled structures DOTATOC and DOTATATE are also agonists (Fig. 3b) [65]. In 2020, Ballal et al. reported ^225^Ac-labeled DOTATATE for 32 patients with GEP-NETs, which showed partial remission in 15 patients and stable disease in 9 [66]. In addition, the phase III trial evaluating the efficacy and safety of ^177^Lu-DOTA-TOC in patients with GEP-NETs is ongoing (NCT03049189), with estimated completion by June 2029. Furthermore, the introduction of SSTRs antagonists, such as LM3, and JR11, is an important advance in the field of SSTRs targeting [63]. Preclinical and clinical studies have shown that SSTR antagonists have better binding ability to SSTR, a higher tumor uptake rate, and can deliver a higher dose of radiation than agonists (Fig. 3c) [67–69]. For example, the antagonist ^177^Lu-DOTA-JR11 had longer tumor retention time and higher tumor uptake compared to ^177^Lu-DOTATATE in patients with NETs [68]. Obviously, the use of radiolabeled SSTR antagonists may offer more successful therapy strategies for NETs compared to agonists and warrant further validation.

**Table 2:** Examples of peptide-based radiopharmaceuticals for cancer therapy.

Target	Radiopharmaceuticals	Cancer	Phase	Ref.
SSTR	^177^Lu-DOTATATE	GEP-NET	Approved	[55]
	^177^Lu-DOTATOC	NENs	III	NCT03049189
	^90^Y-DOTA-TOC	GEP-NET	III	NCT03049189
	^177^Lu-DOTA-JR11	NETs	I/II	NCT02592707
	^161^Tb-DOTA-LM3	NENs	I	NCT05359146
	^213^Bi-DOTATOC	NENs	Clinical	[139]
	^225^Ac-DOTATATE	NENs	Clinical	[66]
	^212^Pb-DOTATATE	NETs	I	NCT03466216
	^177^Lu-DOTA-Peptide 2	SSTR(+)tumors	Preclinical	[140]
	^177^Lu-DOTA-LM3	NENs	Clinical	[141]
	^67^Cu-CuSarTATE	NENs	Preclinical	[142]
GRPR	^177^Lu-AMBA/RM2	Prostate cancer	Clinical	[74, 143]
	^68^Ga/^177^Lu-NeoBOMB1	Solid tumors	I/II	NCT03872778
	^111^In/^177^Lu-SB3	Prostate cancer	Preclinical	[144]
Integrins	^111^In-PSi-iRGD NPs	Prostate cancer	Preclinical	[27]
GRPR/PSMA	^125^I-BO530	Prostate cancer	Preclinical	[56]
Integrin/GRPR	RGD-Glu-[^90^Y-DO3A]-6-Ahx-RM2]	Prostate cancer	Preclinical	[77]
PSMA/GRPR	^177^Lu-DOTA-iPSMA-Lys-BN	Prostate cancer	Preclinical	[145]
GnRH-R	^177^Lu-DOTA-TRPHYD	Prostate cancer	Preclinical	[146]
NK-1R	^90^Y-DOTAGA-SP	Glioma	Clinical	[79]
	^213^Bi/^225^Ac-DOTA-SP	Glioma	Clinical	[80, 147]
	^177^Lu-AuNP-SPTyr8	Glioma	Preclinical	[82]
	^177^Lu-DOTA-SP	Glioma	Preclinical	[148]
Integrins	^177^Lu-AuNP-RGD	Glioma	Preclinical	[149]
SSTR	PEG-LuD-NP	Glioma	Preclinical	[83]
VEGF/Integrins	^177^Lu-Au-NLS-RGD-Aptamer	Glioma	Preclinical	[84]
VEGF/Integrins	iRGD-C6-lys(^211^At-ATE)-C6-^D^A7R	Glioma	Preclinical	[81]
MMP-1	^177^Lu-BCY-B/C/D	MMP-14(+)tumors	Preclinical	[23]
CCK2R	^177^Lu-PP-F11N	MTC	I	NCT02088645
	^177^Lu-DOTA-MGS5	MTC	Preclinical	[150]
	^225^Ac-DOTA-PP-F11N	CCK2R(+)tumors	Preclinical	[151]
CXCR4	^177^Lu/^90^Y-Pentixather	Multiple myeloma	Clinical	[152]
	^177^Lu-DOTA-r-a-ABA-CPCR4	Lymphoma	Preclinical	[153]
Neurotensin	^177^Lu-3BP-227	Solid tumors	I	NCT03525392
	^177^Lu-DOTA-NT	Colon cancer	Preclinical	[154]
Upar	^177^Lu-DOTA-AE105	Colorectal cancer	Preclinical	[155]
MC1R	^177^Lu-DOTA-αMSH-PEG-Cy5-C′dots	Melanoma	Preclinical	[156]
GLP1-R	^177^Lu-DO3A-VS-Cys40-Exendin-4	Insulinoma	Preclinical	[157]

#### Prostate cancer

**Fig. 4:**
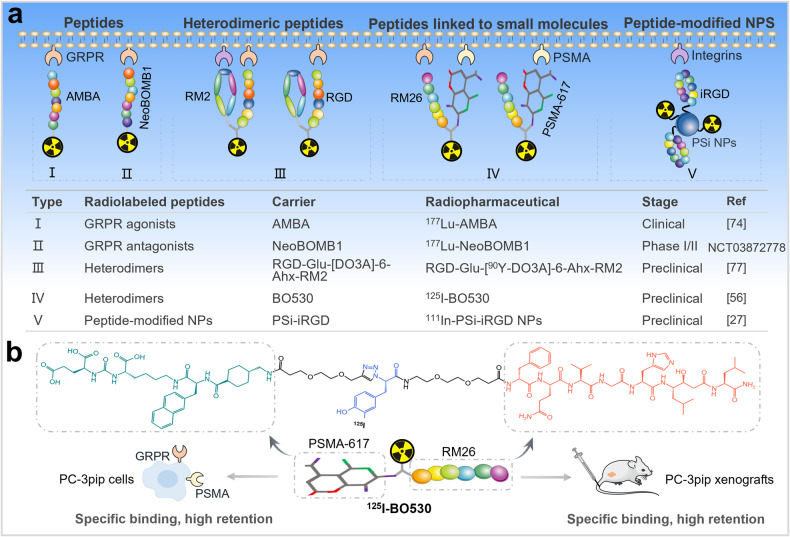
Radiolabeled peptides in prostate cancer therapy. **a** Radiolabeled peptides, heterodimeric peptides, heterodimers of peptides linked to small molecules, and peptide-modified NPs for prostate cancer therapy. **b** Radiolabeled GRPR/PSMA heterodimers for prostate cancer therapy.

Prostate cancer overexpresses specific receptors, including gastrin-releasing peptide receptor (GRPR), prostate specific membrane antigen (PSMA), and integrins, etc. (Fig. 4a) [70]. Radiolabeled bombesin (BBN) analogs exhibit high binding affinity and specificity to GRPR, and its research in the field of GRPR-positive cancer therapy has been thriving [71]. The first radiolabeled BNN analog for therapeutic application is the potent GRPR agonist ^177^Lu-AMBA, which binds to GRPR with high affinity, showing high therapeutic potential [72, 73]. However, a phase I escalation study in patients with metastatic castration-resistant prostate cancer (mCRPC) was discontinued due to severe adverse effects after injection of therapeutic doses of ^177^Lu-AMBA [74]. In addition, multiple studies have shown that radiolabeled GRPR antagonists are better than agonists for cancer therapy [75]. Therefore, preclinical and clinical studies have shifted from GRPR agonists to antagonists. For example, GRPR antagonist ^67/68^Ga/^111^In/^177^Lu-NeoBOMB1 exhibited high GRPR binding affinity, high tumor uptake efficiency, and high in vivo metabolic stability in preclinical studies [76]. A phase I/II study is underway to evaluate ^177^Lu-NeoBOMB1 (NCT03872778), and is expected to be completed in October 2025. Furthermore, in recent years, researchers have been developing radiolabeled heterodimers (Fig. 4a). For example, heterodimeric peptides (RGD-Glu-[^90^Y-DO3A]-6-Ahx-RM2) and peptides linked to small molecules (^125^I-BO530) [56, 77]. Compared with the corresponding monovalent peptide ligands, heterodimers can simultaneously or independently bind to different target receptors, resulting in stronger binding to target cells [22]. In 2019, Abouzayed et al. synthesized the GRPR/PSMA-targeting bispecific heterodimer BO530 [56], a small molecule linked to the BBN-based antagonist RM26 (Fig. 4b). It was shown that ^125^I-labeled BO530 specifically bound to both GRPR and PSMA in vitro with high affinity and cellular retention. Similarly, it was specific to both targets in vivo, has long activity retention in tumors and was cleared from normal organs. Moreover, radiolabeled peptide-modified NPs have been used to study prostate cancer therapy (Fig. 4a) [57]. For example, Wang et al. reported that modification of multifunctional porous silicon NPs (PSi NPs) with iRGD peptide enhanced the uptake and retention of NPs in mice bearing prostate cancer xenografts [27]. Thus, radiolabeled peptides have ushered in a novel prospect for prostate cancer therapy.

#### Gliomas

**Fig. 5:**
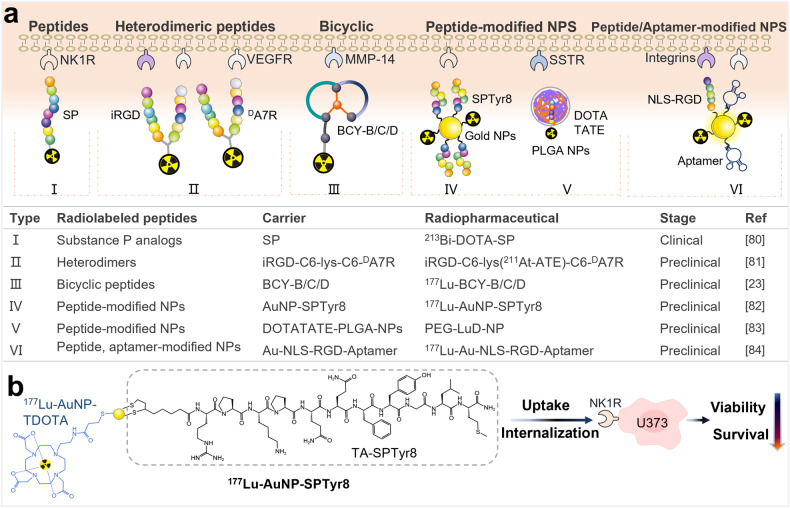
Radiolabeled peptides in glioma therapy. **a** Radiolabeled peptides, heterodimeric peptides, bicyclic peptides, peptide-modified NPs, peptide- and nucleic acid-modified NPs for glioma therapy. **b** Radiolabeled peptide-modified AuNPs for glioma therapy.

Glioma cells express specific receptors and glycoproteins on their surface, such as neurokinin type 1 receptor (NK1R), integrins, and MMP-14, etc. (Fig. 5a) [58]. Substance P is the natural ligand of NK1R, thus radiolabeled substance P analogs can be used for targeting NK1R to treat gliomas (Table 2) [78]. In the phase I study of ^90^Y-DOTAGA-SP in glioblastoma multiforme [79], all patients were well tolerated without acute toxicity. In 2019, ^213^Bi-labeled DOTA-SP was examined in patients with recurrent glioblastoma [80], the median overall survival of patients was 23.6 months, and the median overall survival after recurrence was 10.9 months compares favorably to standard therapy. Additionally, radiolabeled heterodimeric peptides have shown extremely favorable results in preclinical studies for gliomas. For example, in 2022, Liu et al. used radiolabeled heterodimeric peptides iRGD-C6-lys(^211^At-ATE)-C6-^D^A7R targeting both integrins and VEGF receptor for glioma therapy [81], which significantly inhibited tumor growth and prolonged the survival of tumor-bearing mice. Furthermore, radiolabeled bicyclic peptides hold tremendous promise in cancer therapy, and are currently under development. Bicyclic peptides can be synthetically manufactured and bind targets with high affinity and selectivity, providing high tumor penetration and rapid excretion from normal tissue [23]. In 2019, Eder et al. reported radiolabeled bicyclic peptides with subnanometer affinity for MMP-14, which showed selective tumor uptake in a mouse model [23]. Moreover, nanomedicine-based approaches are being used to develop innovative treatment strategies for glioblastoma (Fig. 5) [82–84]. In 2022, Silva et al. designed ^177^Lu-labeled AuNPs carrying substance P derivatives [82], which showed significant radiobiological effects in glioblastoma cells with high cellular uptake and internalization, reduced cell viability and survival, deserving further preclinical evaluation (Fig. 5b). In conclusion, these studies bring hope for new radiolabeled peptide in cancer therapy.

## Radiolabeled nucleic acids in cancer therapy

**Fig. 6:**
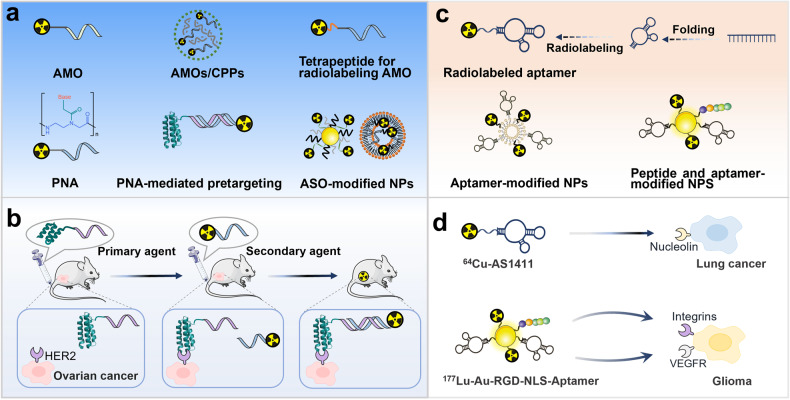
Schematic representation of radiolabeled nucleic acid in cancer therapy. **a** Radiolabeled ASO and some effective strategies to improve ASO delivery. **b** PNA-mediated pretargeting. **c** Radiolabeled aptamer and some effective strategies to improve aptamer delivery. **d** Radiolabeled aptamers as well as radiolabeled aptamer and peptide-modified NPs targeting.

Nucleic acid-based carriers consist of various structural variants. Antisense oligonucleotides (ASO) and nucleic acid aptamers as radiopharmaceutical carriers are very promising (Fig. 6) [24, 85]. Currently, radiolabeled ASOs have achieved a series of successes. In 2016, Kang et al. successfully prepared ^99m^Tc radiolabeled anti-miRNA-155 (AMO-155) oligonucleotide and tested it in vivo in multiple tumor models, demonstrating its high stability and effective delivery [86]. Additionally, more efficient strategies are being developed to improve ASO delivery (Fig. 6a). Firstly, using cell-penetrating peptide-based nanoprobes. For example, in 2021, Yang et al. constructed a novel noncovalent antisense nanoprobe, ^99m^Tc-labeled AMOs/cell-penetrating peptide (CPPs) PepFect6 [87], which showed higher cellular uptake and retention, had low cytotoxicity, high specificity, and sensitivity. Secondly, optimizing the labeling method. In 2022, Chen et al. developed a new tetrapeptide for direct labeling AMO-21 with ^99m^Tc [88]. This labeling approach has high labeling efficiency and stable labeling. Thirdly, the application of nucleic acid analogs antisense peptide nucleic acid (PNA). For example, in 2020, Jiang et al. developed a ^99m^Tc-labeled PNA probe targeting miR-155, which had superior specificity and targeting ability in vitro and in vivo [89]. Fourthly, using PNA-mediated pretargeting. Preclinical studies have shown that this pretargeting is a promising approach for cancer therapy [90, 91]. For example, in 2021, Tano et al. evaluated novel ^177^Lu-labeled PNA probes for the pretargeting (Fig. 6b) [92]. They designed second-generation hybridization probes, the anti-HER2 affibody-PNA primary probe and the complementary secondary probes, and labeled with ^177^Lu. It was shown that the primary agent bound with high specificity and affinity to HER2-expressing cells in vitro, and the secondary hybridization probes that bound with high affinity to the primary agent. In vivo studies showed HER2-specific uptake of all ^177^Lu radiolabeled probes in xenografts in pretargeting. In 2022, Oroujeni et al. reported that the combination of PNA-mediated pretargeting and trastuzumab significantly increased survival in mice bearing HER2-expressing xenografts [93]. Finally, using oligonucleotide functionalized NPs. For example, in 2021, Bavelaar et al. first reported ^111^In-labeled oligonucleotide-functionalized AuNP constructs, which markedly improved the uptake of oligonucleotides and possessed telomerase-specific antiproliferative and cytotoxic effects [94]. In 2022, Ren et al. evaluated the liposome encapsulated ^99m^Tc-labeled ASO probe, which showed good stability and targeting ability [95]. Aptamers are also a highly potential radiopharmaceutical carrier, consisting of functional RNA or single-stranded DNA that can generate unique and diverse tertiary structure to interact with their targets with high affinity and specificity (Fig. 6c) [96]. In this regard, Li et al. prepared the ^64^Cu-labeled oligonucleotide aptamer AS1411 binding to surface nucleolin, which showed reasonable in vivo stability and high binding affinity to cells [97]. Furthermore, aptamer functionalized NPs are also of interest in the delivery of radionuclides [84, 98] (Fig. 6c, d). For example, Bandekar et al. evaluated the therapeutic effect of A10 PSMA aptamer-labeled liposomes loaded with ^225^Ac, which selectively bind, become internalized, and kill PSMA-expressing cells [98]. This may have potential application value in cancer therapy.

## Radiolabeled small molecules in cancer therapy

**Fig. 7:**
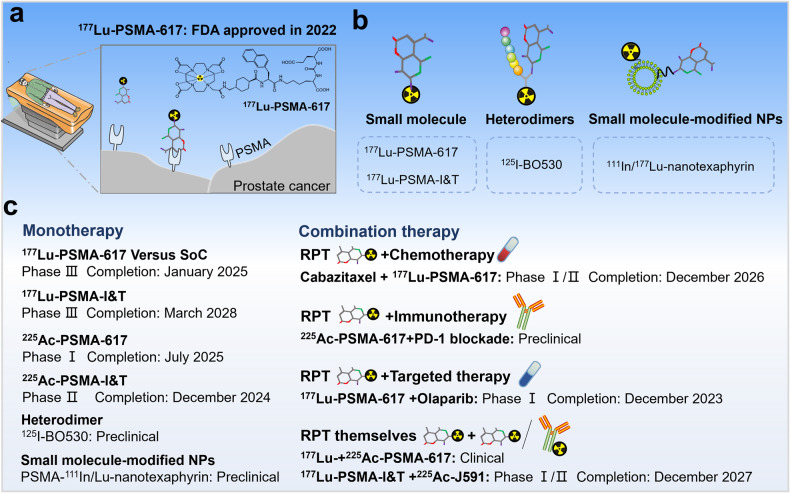
Schematic representation of radiolabeled small molecule in prostate cancer therapy. **a** ^177^Lu-PSMA-617 for the treatment of prostate cancer. **b** Radiolabeled small molecules, heterodimers of small molecules linked to peptides, small molecule-modified NPs for prostate cancer therapy. **c** Novel strategies targeting PSMA for prostate cancer therapy.

Radiolabeled small molecules for cancer therapy are rapidly evolving, particularly targeting PSMA for prostate cancer therapy [99]. In 2022, radiolabeled small molecule ^177^Lu-PSMA-617 was approved by the FDA for the treatment of PSMA-positive mCRPC after androgen receptor pathway inhibition and taxane-based chemotherapy (Fig. 7a) [100]. Additionally, it was reported that radiolabeled heterodimers improved the specificity and accuracy of prostate cancer therapy (Fig. 7b) [56, 101]. For example, Abouzayed et al. synthesized PSMA/GRPR-targeting heterodimer ^125^I-BO530 formed by linking PSMA-617 and peptide, which exhibited targeting specificity and long activity retention both in vitro and in vivo [56]. Small molecule-modified NPs also enhanced the specificity of NPs targeting cancer cells (Fig. 7b). In 2022, Cheng et al. reported that the inclusion of the highly specific PSMA-targeting ligand enabled ^111^In/^177^Lu-nanotexaphyrin to preferential accumulation in PSMA-positive prostate tumors and successfully inhibited tumor growth in a xenograft model [102]. Furthermore, multiple other preclinical and clinical studies are also evaluating novel strategies targeting PSMA for prostate cancer therapy, including monotherapy and combination therapy (Fig. 7c). Many studies of monotherapy are underway. Two phase III trials of ^177^Lu-PSMA-I&T for mCRPC underway to evaluate its safety and efficacy in mCRPC patients (NCT04647526) (NCT05204927). However, a significant number of patients (up to 30%) do not respond or develop resistance to monotherapy with β-emitter ^177^Lu-labeled PSMA-targeted therapy [103, 104], so there is interest in using other radionuclides, notably α-emitter ^225^Ac. A phase I study evaluating the safety of ^225^Ac-PSMA-617 in prostate cancer patients is currently ongoing, with estimated completion by July 2025 (NCT04597411). At the same time, a phase II trial of ^225^Ac-PSMA-I&T for mCRPC is underway, which is expected to be completed in December 2024 (NCT05219500). Several trials are investigating PSMA-RPT in combination with other treatment modalities to improve cancer therapy, including chemotherapy (NCT05340374), immunotherapy [105], and targeted therapies (NCT03874884) [106]. For example, Czernin et al. found that PSMA RPT and PD-1 blockade have synergistic anti-tumor effects [105]. Combination of ^225^Ac-PSMA-617 and anti-PD-1 dramatically improved disease control in a mouse model of prostate cancer compared with either monotherapy. Survival was extended to 51.5 d (control, 28 d; anti-PD-1, 37 d; ^225^Ac-PSMA-617, 32 d; anti-PD-1, 37 d). Besides, RPT targeting PSMA themselves have been combined. For example, co-administration of ^177^Lu-PSMA-617 and ^225^Ac-PSMA-617 [107]. Interestingly, there is a phase I/II clinical trial combining ^177^Lu-PSMA-I&T and the mAb ^225^Ac-J591 for progressive mCRPC, and the estimated study completion date is December 2027 (NCT04886986). Small molecule-based RPT has made significant progress in the past few years, and this therapeutic approach is likely to play an increasingly significant role in the coming years.

## Radiolabeled nanoparticles in cancer therapy

The use of NPs as radiopharmaceutical carriers is developing rapidly, which further improves the therapeutic efficacy of radiopharmaceuticals. NPs can be broadly classified into organic and inorganic NPs (Fig. 1b) [9, 108]. Organic NPs including liposomes, dendrimers, and polymeric NPs. Inorganic NPs including gold NPs, silica NPs, and carbon-based NPs. They have been widely discussed as radionuclide carriers in cancer therapy [9, 109]. In 2022, Huang et al. demonstrated that intravenous injection of ^211^At-labeled AuNPs ^211^At-AuNPs@mPEG significantly inhibited tumor growth in a pancreatic cancer model [110], providing a new framework for the design of NPs suitable for RPT via intravenous injection. In addition, NPs have been effectively functionalized with peptides, antibodies, and aptamers, etc. for specific binding to tumor receptors, and the number of these targeting ligands bound to a NP can be much more than one [111, 112]. For example, González-Ruíz et al. evaluated the therapeutic effect of the aptamer and the peptide-labeled AuNPs loaded with ^177^Lu, which significantly decreased the tumor cell viability in vitro and in vivo, and inhibited tumor progression [84]. In recent years, many preclinical studies have been conducted of radiolabeled NPs and they are expected to play a critical role in cancer therapy.

## Conclusion and perspective

Currently, RPT is emerging as a safe and effective approach to treat many types of cancer. The efficacy of RPT depends on a variety of factors, including the properties of the tumor (permeability, degree of vascularization, and blood flow), the properties of the target (specificity, density, and heterogeneity of expression), the properties of the radionuclide, and the targeting carrier [113]. Therapeutic radionuclides can be reliably attached to carriers and targeted delivery to almost all types of tumors owing to the developed techniques and protocols [114]. We provide an overview of the most commonly employed radiopharmaceutical carrier systems in cancer therapy. Each of these carriers reviewed has advantages and disadvantages, and their characteristics may even change depending on the modifiers or radionuclides used. Therefore, the translation of most carriers into clinics has not progressed as quickly as expected.

Antibody-based radiopharmaceuticals have been widely employed in cancer therapy. However, only two were approved by the FDA. The application of radiolabeled antibodies in solid cancers remains a challenge [3]. It is interesting to note that new discoveries about antibody engineering or radionuclides, and the use of pretargeting strategies are trying to overcome these problems. Radiolabeled peptide is a relatively new and very specific radiopharmaceutical group. There is a growing interest in the development of stable and well-defined novel peptide carrier systems, such as heterodimers and cyclic peptides, which bring hope for new radiolabeled peptides in cancer therapy [23, 81]. However, the available evidence in this regard is limited and further research is needed to fully evaluate its potential. Currently, the development and application of nucleic acids are still in their infancy, and although oligonucleotide-based radiopharmaceuticals have achieved a series of successes in the biomedical field, clinical translation remains slow and many challenges remain to be addressed. Encouragingly, radiolabeled small molecules have made significant progress in mCRPC. ^177^Lu-PSMA-617 has been approved by the FDA, and multiple other clinical trials using different radionuclides and conjugates are underway. Furthermore, antibodies, peptides, nucleic acids, and small molecule-modified NPs are of great interest due to the extraordinary advantages they provided, and are currently being actively investigated. Currently, the scientific community has shown increased interest in developing advanced hybrid radionuclide carriers [84]. Advancement in the field of RPT may further be enabled by using different carrier systems with higher radionuclide loads. However, the safe and effective delivery of radiopharmaceuticals to tumor tissues is still a great challenge in cancer therapy. Thus, there still exists much work to be done in this field in the future, more effective carrier systems remain to be explored and developed. At the same time, combined with chemotherapy, external beam radiotherapy, molecular targeted therapy, and other therapies, RPT has the potential to reach its full therapeutic efficacy in tumor treatment. In short, the field related to radionuclide delivery is still a challenging frontier, with many promising carrier systems waiting to be explored.
